# Genetic structure and dispersal in peripheral populations of a marine fish (Pacific cod, *Gadus macrocephalus*) and their importance for adaptation to climate change

**DOI:** 10.1002/ece3.8474

**Published:** 2021-12-21

**Authors:** Mary C. Fisher, Thomas E. Helser, Sukyung Kang, Wooseok Gwak, Michael F. Canino, Lorenz Hauser

**Affiliations:** ^1^ School of Aquatic and Fishery Sciences University of Washington Seattle Washington USA; ^2^ Resource Ecology and Fisheries Management Division Alaska Fisheries Science Center National Marine Fisheries Service National Oceanic and Atmospheric Administration Seattle Washington USA; ^3^ Fisheries Resources Management Division National Institute of Fisheries Science Busan Korea; ^4^ The Institute of Marine Industry Gyeongsang National University Tongyeong Korea; ^5^ Alaska Fisheries Science Center National Marine Fisheries Service National Oceanic and Atmospheric Administration Seattle Washington USA; ^6^ Present address: School of Environmental and Forest Sciences University of Washington Seattle Washington USA

**Keywords:** gene flow, migration, Pacific cod, peripheral population, population structure, RADseq

## Abstract

Small and isolated peripheral populations, which are often remnants of glacial refugia, offer an opportunity to determine the magnitude and direction of fine‐scale connectivity in high gene flow marine species. When located at the equatorial edge of a species’ range, these populations may also harbor genetic diversity related to survival and reproduction at higher temperatures, a critical resource for marine species facing warming ocean temperatures. Pacific cod (*Gadus macrocephalus*), a marine fish in the North Pacific, has already experienced major shifts in biomass and distribution linked to climate change. We estimated the magnitude and direction of connectivity between peripheral populations of Pacific cod at the southern edge of the species’ range, by conducting restriction site‐associated DNA (RAD) sequencing and individual assignment on fish collected around the Korean Peninsula during the spawning season. Three populations on the western, eastern, and southern Korean coasts were highly differentiated (*F_ST_* = 0.025–0.042) and relatively small (*N_e_* = 433–1,777). Ten putative dispersers and estimates of contemporary migration rates revealed asymmetrical, west‐to‐east movement around the Korean Peninsula, at a higher rate than predicted by indirect estimates of connectivity (*F_ST_*). Allele frequencies at 87 RAD loci were decisively correlated with strong marine temperature gradients between the warmer southern coast and the cooler waters of the eastern and western coasts. Despite relatively small sample sizes, our data suggest asymmetrical dispersal and gene flow, potentially involving adaptive alleles, between peripheral populations inhabiting markedly different thermal regimes. Our study emphasizes the conservation value of peripheral populations in high gene flow marine fish species.

## Graphical abstract

When located at the equatorial edge of a species’ range, small and isolated peripheral populations may harbor genetic diversity related to survival and reproduction at higher temperatures—a critical resource for adaptation to warming oceans. We use restriction site‐associated DNA (RAD) sequencing and individual assignment to reveal west‐to‐east movement of Pacific cod (*Gadus macrocephalus*) between highly differentiated peripheral populations around the Korean Peninsula. A total of 87 RAD loci had allele frequencies decisively correlated with strong marine temperature gradients between the warmer southern population and the cooler eastern and western populations. Our data suggest asymmetrical dispersal and gene flow, potentially involving adaptive alleles, between peripheral populations of a marine fish inhabiting markedly different thermal regimes.

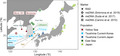

## 1 INTRODUCTION

While many marine species are represented by large population sizes and high gene flow (albeit not lacking genetic structure; Hauser & Carvalho, 2008), they also often include relatively isolated peripheral populations in environmentally distinct habitats (Canino et al., 2010; Knutsen et al., 2003; Ruzzante et al., 2000). Peripheral populations are commonly remnants of refugial populations, left behind after recolonization of current habitats at the end of the last glaciation (Hewitt, 1996; Provan, 2013). Due to their long isolation, these populations exhibit relatively high genetic differentiation (Canino et al., 2010; Maggs et al., 2008) and may be uniquely adapted to their local environment. The importance of the genetic diversity of such populations has been recognized for some time (Hampe & Petit, 2005; Hewitt, 1996), but their potential contribution to adaptive changes in core populations via gene flow of beneficial alleles is still relatively unknown (Provan & Maggs, 2012), especially in marine fishes of commercial importance.

A central question in resolving the significance of peripheral populations for the adaptation of populations at the core of a species’ distribution is the extent and direction of dispersal and gene flow. With next‐generation sequencing techniques, the isolation, old age, and small size of peripheral populations affords the opportunity to directly estimate connectivity via individual assignment. Individual assignment through next‐generation sequencing has been successfully applied to peripheral populations of commercially important species in the Baltic Sea (Johannesson & Andre, 2006), Atlantic herring in Norwegian fjords (Bekkevold et al., 2015), and yelloweye rockfish in the Puget Sound (Andrews et al., 2018). Such fine‐scale data on the magnitude and direction of connectivity are needed to address critical knowledge gaps in commercially important species, from the potential spread of beneficial alleles, which may confer enhanced resilience under climate change, to the identification of source and sink populations for sustainable fishery management.

Pacific cod (*Gadus macrocephalus*) around the Korean Peninsula provide an excellent opportunity to explore connectivity in peripheral populations, and its significance for core populations in the center of the species’ range. The interconnected marginal seas of the northwest Pacific are characterized by a complex glacial history (Ni et al., 2013) and strong environmental gradients (Rebstock & Kang, 2003). Two microsatellite studies (Gwak & Nakayama, 2011; Kim et al., 2010) reported genetic differentiation among Pacific cod in South Korean waters that was over an order of magnitude higher than was found across the entire North American west coast (Cunningham et al., 2009; Spies, 2012). Although putative population boundaries differed between the two microsatellite studies (Figure S1), both describe levels of genetic divergence suitable for individual assignment tests by restriction site‐associated DNA (RAD) sequencing (Drinan et al., 2018).

Pacific cod in Korean waters persist at the southern edge of the species’ western Pacific distribution, spawning at the highest temperatures observed for the species (5–9°C, in contrast to 1–5°C in the Bering Sea; Gustafson et al., 2000). Water temperatures and salinity are particularly high along the southern Korean coast due to the influence of the Tsushima Warm Current (Figure S2; Chang et al., 2004). More northern Pacific cod populations are already experiencing increased mortality from climate‐related water temperature anomalies that fall within the current thermal spawning niche around the Korean Peninsula; for example, the 2014–2016 North Pacific marine heatwave led to mass mortalities of Pacific cod in the Gulf of Alaska when water temperatures rose above 8°C (Barbeaux et al., 2020). Alleles in peripheral Korean populations that convey adaptation to higher water temperatures during spawning and early life history stages may allow more northern populations to persist in warming conditions (evolutionary rescue; Carlson et al., 2014), but only if there is sufficient gene flow across populations (Bell & Gonzalez, 2011).

More precise estimates of population connectivity are also highly relevant for Pacific cod fishery managers, who have been working toward stock recovery in South Korean waters since commercial catches reached historic lows in the 1990s (Figure S3; Kim et al., 2010; Lee & Rahimi Midani, 2014). Pacific cod undertake considerable annual migrations to aggregate at winter spawning grounds (Rand et al., 2014; Shimada & Kimura, 1994), which can be distinguished by genetic and phenotypic differences (Gustafson et al., 2000). As most fisheries are spatially managed, it is of vital importance for managers to account for variation in population boundaries that may arise from seasonal spawning migrations. Individual assignment tests and mixed stock analyses would be powerful tools in these efforts (Dahle et al., 2018).

Our study builds on previous microsatellite research (Gwak & Nakayama, 2011; Kim et al., 2010) to accomplish three objectives: (i) to resolve the spatiotemporal stock structure of Pacific cod around the Korean Peninsula, (ii) to identify dispersers between populations to ascertain the magnitude and direction of gene flow, and (iii) to detect evidence for selective differentiation among these populations. We used thousands of SNPs from RAD sequencing and a more expansive set of samples than prior studies, including within‐ and between‐year temporal replicates, to achieve these aims. We also estimated effective population sizes to establish any effects of recent reductions in abundance. We then considered our results in the context of phylogeographic and contemporary environmental drivers of genetic divergence, and the implications of our study for fisheries management and for the potential spread of beneficial alleles in the northwest Pacific.

## 2 MATERIALS AND METHODS

### 2.1 Sample and data collection

**FIGURE 1:**
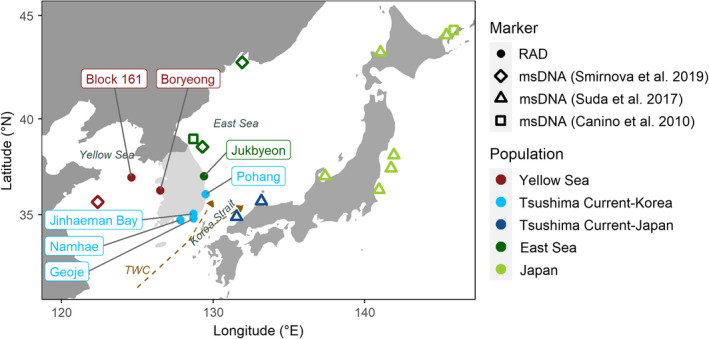
Map of sampling sites around the Korean Peninsula used for this study (labeled points), augmented with results from previous genetic analyses in the surrounding region (Canino et al., 2010; Smirnova et al., 2019; Suda et al., 2017). Point coloration represents the putative populations of each site according to the genetic structure described by RAD loci (this study) and microsatellite DNA (msDNA; previous studies). The approximate path of the Tsushima Warm Current (TWC), with primary branching in the Strait, is traced by the dashed orange arrows

A total of 322 fin clips and tissue samples were collected from 11 aggregations of Pacific cod (hereafter “collections”) at 7 known spawning sites around the Korean Peninsula (Figure 1; Table 1). Sampling was conducted during the spawning season from December to March. Maturity stage was estimated by comparing each sample’s total length to sex‐specific 50% length‐at‐maturity estimates from the East and Yellow Seas (Lee et al., 2016), and calculating the gonadosomatic index (100 × gonad/total weight) when weight data were available. Immature individuals were flagged but retained in our analysis (Table 1).

**TABLE 1:** Pacific cod samples collected from aggregations at known spawning sites (“collections”) around the Korean Peninsula

Coast	Site	Season	No. Samples (Mature)	Mean SNPs Genotyped	*m*	*F_IS_*	*H_E_*	*H_O_*	*N_e_*
West	Boryeong*	2007	21 (*20*)	5735 (99%)	0	−0.020	0.186	0.194	1,777
	Block 161^+^	2015	25 (*25*)	5578 (96%)	0	−0.006	0.183	0.188	690
South	Jinhaeman Bay*	2007, Dec.	39 (*39*)	5558 (96%)	0	0.010	0.171	0.172	188
		2007, Feb.	34 (*NA*)	5512 (95%)	0	0.009	0.173	0.174	771
	*South (Pooled)*	*2007*	73 (*NA*)			0.010	0.174	0.173	443
	Geoje^+^	2013	28 (*28*)	5589 (99%)	0.03	0.009	0.172	0.175	630
		2014	22 (*22*)	5742 (96%)	0.04	−0.098	0.182	0.203	1,044
	Namhae^+^	2014	11 (*11*)	5756 (99%)	0	−0.028	0.172	0.186	–
	Pohang^+^	2014	31 (*24*)	5751 (99%)	0.07	−0.060	0.176	0.190	1,617
	*South (Pooled)*	*2014*	64 (57)			−0.064	0.181	0.195	1,576
East	Jukbyeon*	2007	32 (*0*)	5533 (95%)	0.125	0.065	0.184	0.175	1,664

Note

Maturity is based on sex‐specific 50% length‐at‐maturity estimates. Mean SNPs genotyped reports the number and proportion of RAD loci genotyped across all fish in the given collection. Population‐level statistics (*F_IS_*, *H_E_*, *H_O_*, *N_e_*) were calculated after removing any migrants (*m*) identified in Structure and a principal component analysis (PCA; see Figure 3). Samples were pooled across sites within seasons to calculate population‐level statistics for the southern coast, reflecting negligible differentiation. Samples provided by *Gyeongsang National University (Gwak & Nakayama, 2011 *) and* ^+^ *Republic of Korea National Institute of Fisheries Science*.

DNA was extracted from fin clip and tissue samples using DNeasy 96‐well Blood & Tissue kits (Qiagen Inc.), then quantified with Quant‐iT PicoGreen dsDNA Reagent (Invitrogen). RAD libraries were prepared according to Baird et al. (2008) and Etter et al. (2011), with modifications developed for North American Pacific cod samples by Drinan et al. (2018). Quality filtering and demultiplexing of raw sequences, *de novo* construction of a reference database of RAD loci, SNP discovery, and genotyping, was completed using a combination of the Stacks v1.44 pipeline (Catchen et al., 2011, 2013), Bowtie (Langmead et al., 2009), and NCBI’s Basic Local Alignment Search Tool, BLAST (Altschul et al., 1990), according to the procedures outlined in Drinan et al. (2018) and Brieuc et al. (2014). Final filtering removed loci with a minor allele frequency (MAF) <0.05 in every Pacific cod collection, with greater than 30% missing data across all collections, and which did not conform to Hardy–Weinberg equilibrium (HWE). Individuals missing more than 30% of genotypes or showing evidence of cross‐sample contamination (putative relatedness to other individuals and heterozygosity *H_O_* > 0.25) were also removed. We used Genepop v4.2 (Rousset, 2008, 2014) to estimate observed and expected heterozygosity, *F_IS_*, and to perform exact tests for the identification of loci significantly out of HWE according to Fisher’s combination of probabilities (Sokal & Rohlf, 1995). Putative genetic relationships were estimated using the maximum‐likelihood method in ML Relate (Kalinowski et al., 2006).

### 2.2 Population analysis

Genepop v4.2 was used to estimate locus‐specific *F*‐statistics (*F_ST_*, *F_IS_*, *F_IT_*), *F_IS_*, and observed heterozygosity (*H_O_*) per collection and pairwise *F_ST_* between all collections (Weir & Cockerham, 1984). We tested for significant population differentiation using Fisher’s exact probability test on the distribution of diploid genotypes (Rousset, 2008) and a permutation test on pairwise *F_ST_* in the R package *strataG* (Archer et al., 2016), then adjusted *p*‐values using the Bonferroni correction for multiple testing. We also recalculated pairwise *F_ST_* after removal of loci putatively under selection; these values are reported in the Supplemental Information (Table S1). We used a Wilcoxon rank sum test to identify significant changes in *F_IS_* and *H_O_* between spawning seasons, and for significantly differentiated populations sampled across multiple seasons.

Principal component analysis (PCA) conducted with the R package *adegenet* (Jombart, 2008) was used to visualize genetic differences between samples. The number of genetically distinct populations was determined using Structure v2.3.4, run with a burn‐in period of 50,000, followed by 100,000 Markov chain Monte Carlo (MCMC) replicates (Porras‐Hurtado et al., 2013; Pritchard et al., 2000). The number of populations (*K*) was varied from 1 to 9, and the optimum number was determined using the mean log‐likelihood (Evanno et al., 2005).

Effective population size was estimated using the linkage disequilibrium method in NeEstimator v2.0 (Do et al., 2014); we report results using a minor allele frequency cutoff of 0.05 (Drinan et al., 2018). This naive effective population size was then corrected for downward bias from physical linkage between loci using the least‐squares regression of *ln(chr)* (Waples et al., 2016), assuming that Pacific and Atlantic cod have the same number of chromosomes (23; Tørresen et al., 2017). We conducted the same correction for *N_e_* estimates from Table 1 of Drinan et al. (2018) for comparison.

### 2.3 Individual dispersal and migration

Group membership probabilities for individuals dispersed outside of their putative source populations were calculated in GeneClass (Cornuet et al., 1999; Piry et al., 2004). Resident individuals (i.e., non‐dispersers) were assigned to populations of origin using the training, holdout, and leave‐one‐out method (Anderson et al., 2008; Anderson, 2010; Benestan et al., 2015) implemented with *gsi_sim* in the R package *assigner* v0.5.0 (Gosselin et al., 2016). We also estimated contemporary migration rates and direction (within the last two generations) using a Bayesian identification of first‐ and second‐generation immigrants in BA3‐SNPs (Mussmann et al., 2019; Wilson & Rannala, 2003). Acceptance rates of the MCMC chain were optimized to 28%–50% by adjusting mixing parameters with BA3‐SNPs‐autotune (Mussmann et al., 2019), and convergence confirmed by log probability traces in Tracer 1.7 (Rambaut et al., 2018). Final estimates were obtained in a run with 15 million MCMC iterations, with a burn‐in period of 5 million and a sampling interval of 100 iterations.

### 2.4 Outlier loci detection and alignment

We identified putatively adaptive loci using two outlier tests and by testing for gene‐environment associations. Candidate outlier loci were identified with both BayeScan v2.1 (Foll & Gaggiotti, 2008) and OutFLANK (Whitlock & Lotterhos, 2015) to account for variation in the relative power of each test under different population structure (Whitlock & Lotterhos, 2015). We used the default settings and a false discovery rate of 0.05 to identify candidate outliers in Bayescan v2.1, varying the prior odds for the neutral model stepwise from 10 to 100, 1,000, and 10,000. In OutFLANK, we set the threshold for detection of outlier loci at *q* = 0.05 and trimmed 5% of *F_ST_* values from the right and left tails of the distribution. We then used Bayenv2 (Coop et al., 2010; Günther & Coop, 2013) to test for significant correlation between allele frequencies and four water temperature variables that best represented the Tsushima Warm Current temperature gradient on the southern coast: mean and maximum sea surface temperature, and mean and minimum temperature at maximum depth. Water temperature estimates at each sampling site were obtained from the Bio‐Oracle and Bio‐Oracle 2 databases (Assis et al., 2018) using the R package *sdmpredictors* (Bosch et al., 2017). By accounting for neutral correlations of allele frequencies, including those arising from migration and genetic drift, the Bayenv2 analysis of gene–environment associations is more robust to demographic population structure (Günther & Coop, 2013). We retained correlations with a Bayes factor >100 (“decisive support”; Kass & Raftery, 1995).

The Pacific cod genome has not been assembled, and so to explore whether loci putatively under selection co‐localized with annotated genes, candidate outlier loci (all identified by OutFLANK; Bayescan prior odds ≥100) and loci decisively correlated with temperature gradients were aligned to the Atlantic cod genome, *gadMor2* (Tørresen et al., 2017), using Bowtie2 (Langmead & Salzberg, 2012). We filtered alignments for a mapping quality greater than 10, and matched annotations that fell within gene regions using bedtools v2.24 (Quinlan & Hall, 2010).

## 3 RESULTS

**FIGURE 2:**
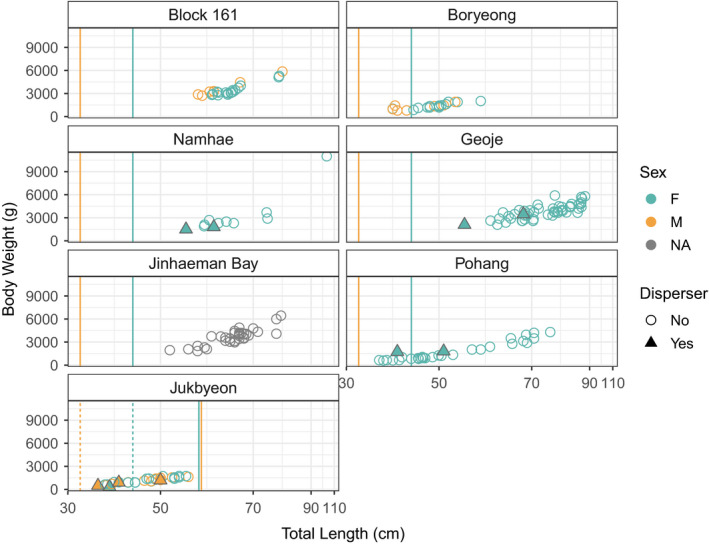
Distributions of sex‐specific total length versus body weight at each sampling site. Both sampling years for Geoje are included, but only the early spawning fish from Jinhaeman Bay (Dec. 2007 collection) were measured. Sex data were not available for Jinhaeman Bay samples. Solid vertical lines indicate the appropriate sex‐specific 50% length‐at‐maturity estimate for the East Sea or the Yellow Sea / southern coast. Triangle data points indicate putative dispersers, which are shown by the spawning site where they were sampled. At Jukbyeon, the 50% length‐at‐maturity estimate for the immigrants’ source population (western and southern coasts) differed from the estimate at that site, and so the 50% length‐at‐maturity estimate for the putative source population is depicted with dashed vertical lines

The final dataset consisted of 5,804 RAD loci and 243 individuals (Table 1). Most individuals (94%) were above sex‐specific 50% length‐at‐maturity at all sampling sites except Jukbyeon (Table 1; Figure 2). Average *F_IS_* over loci remained close to zero for every collection, with a global locus *F_IS_* of −0.009, *F_ST_* of 0.022, and *F_IT_* of 0.013.

### 3.1 Strong genetic breaks over small spatial scales

Pairwise *F_ST_* between collections on different coasts was high (up to *F_ST_* = 0.042) and significant, with no significant genetic differentiation between collections within coastal regions or between temporal replicates (Table 2). Two within‐coast comparisons were significant only with the permutation test: the collection of fish sampled at Geoje in the 2014 spawning season compared to the remainder of the southern samples, and the comparison between the offshore/inshore western coast collections.

**TABLE 2:**
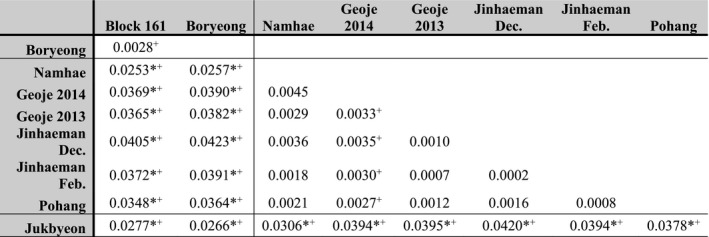
Pairwise *F_ST_* between collections, including temporal replications

Note

Lines segregate sites from different coasts (west, south/southeast, and east). Statistically significant differentiation is indicated with (*) for Fisher’s exact test and (+) for strataG’s permutation test.

**FIGURE 3:**
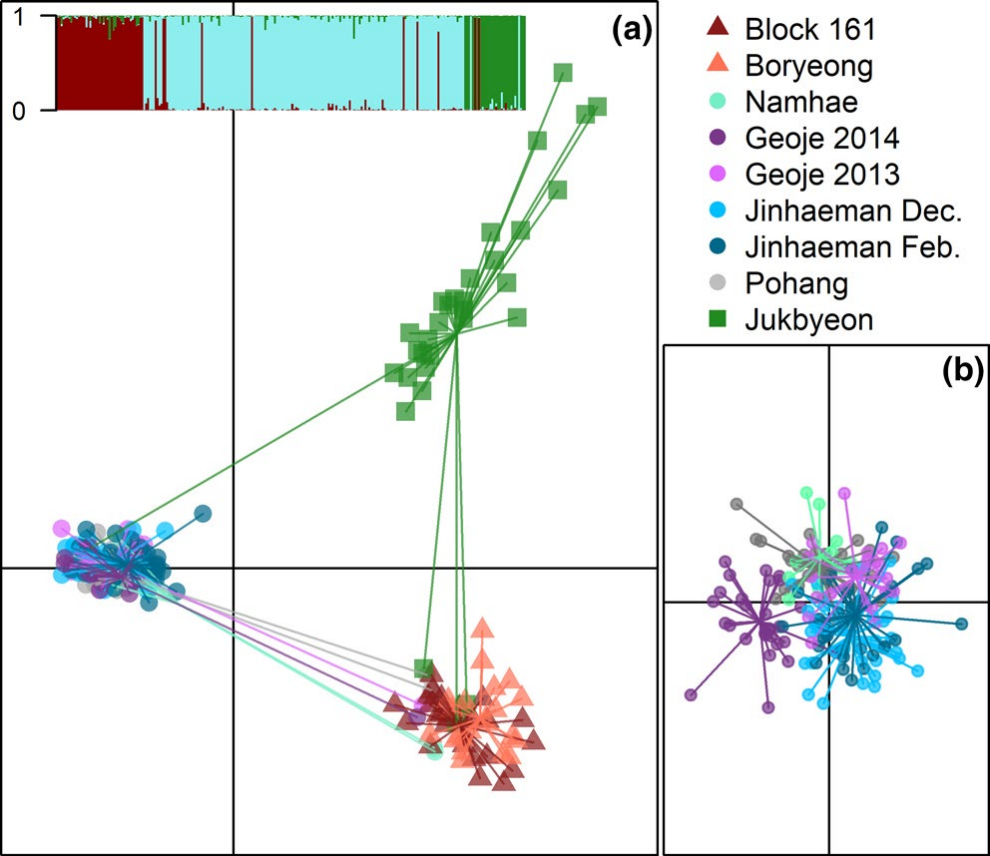
Principal component analysis of all samples (a) and discriminant analysis of principal components for southern samples only (b). The first and second principal components of the principal component analysis (PCA) explained 3.09% and 1.65% of the variation in the data. Inset (a) shows proportion of membership to the western (red), southern (blue), and eastern (green) coast populations according to Structure for each individual, ordered by collection from west to east. Note that individuals which cluster with a different population in the principal component analysis display a majority membership in a different population than their neighbors in the Structure inset

Bayesian clustering in Structure suggested three genetically distinct populations within this dataset: the western coast, the southern coast and the eastern coast (Figure 3a inset). This delineation was supported by the three distinct clusters of the principal component analysis (PCA), composed of the western (Block 161, Boryeong), eastern (Jukbyeon), and south/southeastern (Namhae, Geoje, Jinhaeman Bay, Pohang) sampling sites (Figure 3a). A discriminant analysis of principal components (DAPC) with only individuals collected from the southern coast showed minimal spatiotemporal structure with slight separation of the Geoje 2014 collection, reflective of low pairwise *F_ST_* (Figure 3b). Similar clustering was observed when the PCA and DAPC were constructed using only loci putatively under selection (Figure S4).

### 3.2 Individual dispersal and assignment

Ten individuals were genetically distinct from the coastal population from which they were sampled (Table S2). In the PCA, three fish (2 males, 1 female) caught at Jukbyeon on the eastern coast clustered with the western collections, and one female with the southern collections; six females sampled from the southern coast clustered with the western collections (Figure 3a). Structure and GeneClass confirmed the genetic origins suggested by the PCA. In Structure, the inferred proportional ancestry for all 10 dispersers was >80% for the coastal region with which the individuals clustered (Figure 3a inset; Table S3), and in GeneClass all dispersers had 100% assignment probabilities to the coastal region with which the individuals clustered (Table S4). BA3‐SNPs identified 9 of the 10 dispersers as first‐generation immigrants (posterior probability 1.0; Table S4), and one individual sampled at Geoje as a second‐generation immigrant with west coast ancestry (posterior probability 1.0). In addition, BA3‐SNPs identified a female sampled at Jukbyeon as a second‐generation immigrant with south coast ancestry (posterior probability 1.0). This individual clustered with the sampled eastern population in the PCA (Figure S4), and had the greatest inferred ancestry from the sampled population in Structure (78.8%; with 18.3% and 2.8% associated with the southern and western coast populations, respectively). These two second‐generation hybrids strongly suggest that dispersal resulted in some gene flow.

Of the five first‐generation immigrants sampled at the southern sites, and the four sampled at Jukbyeon, all but two were above the 50% length‐at‐maturity estimate for their putative source population (Figure 2). The second‐generation immigrant sampled at Geoje was above 50% length‐at‐maturity for both the sampled and putative source populations. Of the four dispersers with recorded gonad weights, two had gonadosomatic indices (GSIs) in the 75th percentile for non‐immigrant individuals at their respective sampling sites, and one each in the 50th and 25th percentiles (Figure S5).

We tested assignment success of all non‐migrant individuals to collection and population (per PCA and Structure) of origin, using a reduced marker set. Overall assignment success to population of origin reached 100% using only 100 markers, and almost 90% with only 10 markers (Figures S6 and S7).

### 3.3 Migration rates

All pairwise eastward migration rates per generation were almost an order of magnitude higher than pairwise westward migration rates (inferred posterior means 1.4%–3.8% v. 0.2%–0.7%, respectively; Table S5, Figure S8). According to BA3‐SNPs, migration from the west to the east coast of the Peninsula was the greatest among all pairwise comparisons (3.8 ± 1.8%), with the second highest migration rate from the south to the east coast (2.6 ± 1.6%). The least migration over the last two generations was from the east to the south coast of the Peninsula (migration rate of 0.2 ± 0.2%). 95% confidence intervals for all pairwise westward migration rates overlapped with zero (Table S5, Figure S8).

### 3.4 Small effective population sizes

Physical linkage between loci caused a downward bias of approximately 27.4% in the naive estimates of effective population size (*N_e_*). Removal of putative immigrants resulted in a 3.4% (Geoje 2014–2015) to a 461% (Pohang) increase in effective population size (Table S6).

**FIGURE 4:**
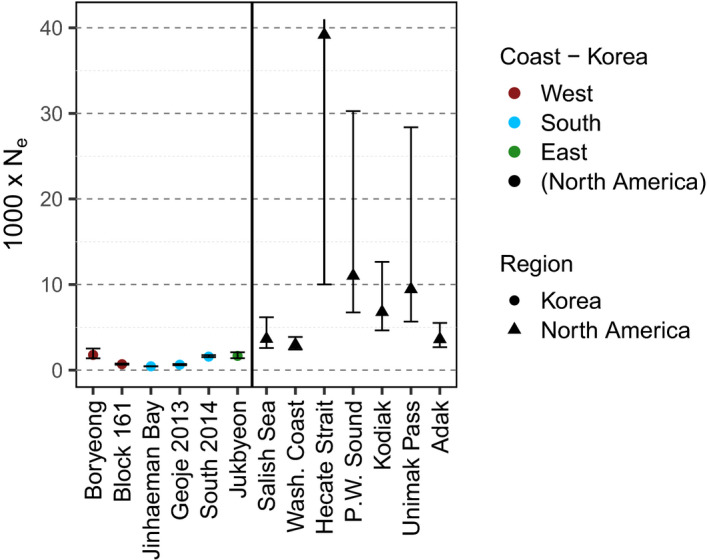
Effective population size (*N_e_*) corrected for physical linkage with 95% confidence intervals for the northwestern Pacific (left panel) and the northeastern Pacific (right panel; Drinan et al., 2018). Collections along the southern coast of the Korean Peninsula (left panel; blue) are pooled for the 2007–2008 (Jinhaeman Bay: Jinhaeman Dec. and Feb.) and 2014–2015 (South 2014: Pohang, Namhae, Geoje 2014) spawning seasons to reflect their belonging to a single genetically distinct population. The upper bound of the Hecate Strait confidence interval was infinite

Effective population size for each collection ranged from *N_e_* = 443 at Jinhaeman Bay (Dec. 2007–2008) to *N_e_* = 1,777 at Boryeong when the lowest minor allele frequency used was 0.05 (Table 1; Figure 4). All upper confidence limits were finite and below *N_e_* = 2,000 (Figure 4). Effective population size of pooled southern coast collections from the same spawning season increased from 443 in the 2007 season to 1576 in 2014 (Table 1; Figure 4). We observed a corresponding and significant increase in observed heterozygosity, and decrease in *F_IS_*, over time (Table S7).

### 3.5 Candidate loci under selection

OutFLANK found significant evidence for selection at 10 loci (Table S8), whereas Bayescan identified 100, 29, 12, and 6 candidate outlier loci when prior odds of the neutral model were set to 1 in 10, 100, 1,000, and 10,000, respectively. Nine candidate outlier loci were identified by both programs (Figure S9). Bayenv2 identified 87 loci with allele frequencies that were decisively correlated (Bayes factor >100) with one or more of the four temperature variables, 74 of which had the most extreme allele frequencies in the warmer southern, rather than the cooler eastern or western, population (Table S9). Forty of the 87 loci identified with Bayenv2 were also identified as candidate outlier loci by OutFLANK or Bayescan (prior odds of 1 in 10; Figure S9).

Sixty‐three unique loci identified by Bayescan (prior odds ≥ 100), OutFLANK, and/or Bayenv2 aligned to sequences in the Atlantic cod genome with a mapping quality >10. Fifty‐eight of these (including 25 candidate outlier loci and 45 temperature‐associated loci) aligned within annotated protein‐coding regions of the Atlantic cod genome, 42 of which coded for proteins of known function (Tables S10 and S11).

## 4 DISCUSSION

We analyzed over 5,800 RAD loci in 243 individuals across four spawning seasons to quantify the magnitude and direction of connectivity between peripheral populations of Pacific cod around the Korean Peninsula. We first identified three genetically distinct populations along the three coasts of the Korean Peninsula, consistent with findings from microsatellite DNA reported by Gwak and Nakayama (2011). The remarkably high genetic differentiation among these populations (lowest *F_ST_* ≈ 0.02) made it possible to observe 10 individuals representing unidirectional, west to east dispersal; disequilibrium‐based estimates of contemporary population connectivity reflected asymmetrical migration eastward around the Korean Peninsula. The potential adaptive significance of this dispersal was indicated by 147 putatively adaptive loci, 87 of which were correlated with temperature and 58 of which could be annotated using the Atlantic cod genome. Our results support the use of peripheral populations in studies of population connectivity in high gene flow species, and the importance of such populations at the southern range edge as a potential source of adaptive genetic diversity.

### 4.1 Demographic history, regional oceanography, and local adaptation form and maintain peripheral populations

Our RAD sequencing results add to previous microsatellite studies (Canino et al., 2010; Gwak & Nakayama, 2011; Kim et al., 2010; Smirnova et al., 2019; Suda et al., 2017) to provide a more complete picture of Pacific cod population structure in the marginal seas of the northwestern Pacific (Figure 1). Pacific cod around the Korean Peninsula form three genetically distinct populations, as demonstrated by our results and by Gwak and Nakayama (2011): one along the western coast in the Yellow Sea, one on the southern coast within the Tsushima Warm Current, and one on the northeastern coast in the East Sea. Previous microsatellite studies indicate that these three Korean populations represent small peripheral populations that are relatively isolated from those in Japanese waters, and those further north toward the core of the species’ distribution. Canino et al. (2010) and Smirnova et al. (2019) identified a genetic break in the East Sea dividing populations in northeast Korea from northeast Japan; the homogeneity of cod around Japan (Suda et al., 2017) suggests the same genetic break separates populations along the western Japanese coast from those in northeast Korea. However, two divergent Japanese collections stem from the warm Tsushima Warm Current (Suda et al., 2017) and thus could be part of the southern Korean population identified here (Figure 1), although there is no direct comparison of samples across the Korea Strait.

The remarkably high levels of genetic differentiation between the three populations of Korean cod are surprising for a marine fish over such short distances. The formation and maintenance of such sharp genetic breaks between peripheral populations may be attributed to a combination of the species’ phylogeographic history in the region and environmental gradients produced from present‐day oceanographic patterns. Pacific cod around the Korean Peninsula were likely isolated in at least two glacial refugia in the late Pleistocene: (1) the Okinawa Trough, formed as sea levels fell in the Yellow Sea and East China Sea, and (2) a semi‐isolated inshore basin in the current East Sea (Park et al., 2000; Wang, 1999). The phylogeography of other marine fauna in this region also reflect ice‐age separation in the independent refugia of the East Sea and the Okinawa Trough (Ni et al., 2013; Xu et al., 2009). Since Pacific cod expanded into the northwest Pacific 102–500,000 years ago (Canino et al., 2010), this late Pleistocene separation would have been the last of several periods of population contraction and expansion.

Historical differentiation may be maintained by contemporary oceanographic currents, which give rise to strong thermal gradients (Figure 1; Figure S2). The Tsushima Warm Current and East Korean Warm Current carry warmer, more saline waters along the southern and southeastern Korean coast (Chang et al., 2004), while the North Korean Cold Current and Korean Coastal Current are associated with colder temperatures on the eastern and western coasts, respectively (Chang et al., 2004; Hwang et al., 2014). Temperature differences are notable; February nearshore temperatures have been recorded as 0–4°C along the eastern coast (Chung et al., 2013), compared to 7–9°C in Jinhaeman Bay on the southern coast (Gwak et al., 2012). Since sea surface temperature during and after the spawning period are important for Pacific cod reproduction, the warmer waters of the southern coast may maintain high genetic divergence by imposing selective pressure during spawning and early development (Gwak & Nakayama, 2011). Most loci with allele frequencies decisively correlated with thermal gradients had unique allele frequencies in the southern population (Table S9); approximately half of the putatively adaptive loci which aligned within known protein‐coding regions were associated with reproduction and early development (Table S11). For example, two loci with allele frequencies correlated with mean sea surface temperature and minimum temperature at depth aligned within a gene coding for KMT2a/b, a methyltransferase that plays a role in oocyte growth and is required during a period of global transcriptional silencing that precedes oocyte survival and normal zygotic genome activation (Andreu‐Vieyra et al., 2010; Dahl et al., 2016). Adaptations which impact fitness and survival during early life history stages confer a selective advantage at a “critical period” of high mortality in marine fishes (Hjort, 1914). It is possible that other co‐occurring environmental gradients between these populations (e.g., salinity) contribute to the adaptive differentiation we observed; additional gene–environment analyses using our dataset, or a more expanded dataset including samples from other populations in the region, could identify key and/or confounding environmental variables driving adaptation in this species.

The three peripheral populations on the Korean Peninsula also had low effective population sizes, well within the range where genetic drift can reduce standing diversity and contribute to high genetic divergence. Our estimates of effective population size were all within a narrow range of *N_e_* = 188–1,777. While these estimates are very low for a marine species supporting commercial fisheries, they are supported by (i) their consistency across different samples, (ii) the fisheries catch statistics showing a depletion of the stock 1950–2005 (Figure S3), (iii) the lower expected heterozygosity compared to Drinan et al. (2018), (iv) the lower heterozygosity of the southern population compared to the eastern and western populations (Table 1), and (v) the low mtDNA haplotype diversity in Korean waters, which is the lowest of the entire sampled Pacific cod range (Canino et al., 2010). Our *N_e_* estimates were even lower than those of the Salish Sea population of Pacific cod (*N_e_* = 2,861), another peripheral population in the eastern north Pacific, which is listed as a Species of Concern by the National Oceanic and Atmospheric Administration (Drinan et al., 2018).

High genetic differentiation and relatively small population sizes, as reported in this study, along with low genetic diversity (in northwestern Pacific cod, mtDNA diversity; Canino et al., 2010; Suda et al., 2017) and harsher conditions (e.g., high temperatures), are predicted by the center–periphery hypothesis (Pironon et al., 2017) for peripheral populations that persist at the geographic and ecological margins. This biogeographic paradigm seeks to explain the distribution of genetic and demographic variability across species ranges, and the evolution of range margins (Pironon et al., 2017). However, the isolation of peripheral Korean cod populations contradicts the center–periphery hypothesis, which postulates that gene swamping from the core to the periphery prevents local adaptation to marginal habitats (Pironon et al., 2017). The relative isolation of Korean cod populations—in addition to their age likely predating the last glaciation, their asymmetrical gene flow, and unusual environmental conditions—instead promotes local adaptation. This may allow them to persist longer in a changing climate than they would if they were connected to the center of the distribution (Nadeau & Urban, 2019). Indeed, South Korean cod spawn at high water temperatures equivalent to those that led to the collapse of the Gulf of Alaska Pacific cod population (Barbeaux et al., 2020; Laurel & Rogers, 2020). Gene flow between peripheral and core populations is therefore a crucial factor in the determination of range margins, especially in a changing climate (Nadeau & Urban, 2019).

On the other hand, the isolation of Korean cod populations and near‐zero east‐to‐west migration rates suggests that they will not benefit from demographic or genetic rescue if they collapse because of overfishing, pollution, or climate change. Management should therefore be very conservative, as the loss of these populations would cause an irreversible range contraction and loss of neutral and adaptive genetic diversity in the species. Population‐specific monitoring could be facilitated by individual assignment, which we show to be highly accurate with few loci.

### 4.2 Directional connectivity and export of adaptive differentiation

Ten individuals were identified as between‐population dispersers, all moving counterclockwise (west to east) around the Korean Peninsula. This eastward dispersal is in accordance with the asymmetrical migration rates estimated with BA3‐SNPs; migration eastward around the Peninsula over the last two generations was on average five‐fold greater than westward migration. Although the number of dispersers we observed was relatively small, the magnitude of asymmetry presents a strong case for highly directional west‐to‐east connectivity. Gwak and Nakayama (2011) found similar evidence for eastward dispersal around the Korean Peninsula: one of their sampling sites on the northeast coast was a mixed‐composition aggregation of Pacific cod originating from both eastern and southern coast spawning populations. The direction of dispersal in this study follows a series of strong currents around the Korean Peninsula (Chang et al., 2004), suggesting that larval advection from natal spawning grounds may be a predominant force (similar to walleye pollock, *Gadus chalcogrammus*, in the Bering Sea; Duffy‐Anderson et al., 2017). On the other hand, spent Pacific cod have been documented moving from the southern coast to deeper waters in the East Sea, providing evidence for dispersal in the same direction at later life stages (Lee et al., 2015).

Unidirectional dispersal from peripheral populations into core populations has been reported among northeastern Pacific cod (Drinan et al., 2018) and yellow‐eye rockfish (Andrews et al., 2018). Although recent otolith microchemical analyses conducted on the same set of Pacific cod samples for several of our study sites (including dispersers collected at Pohang, Namhae) did not support this dispersal pattern, instead suggesting limited dispersal out of the Yellow Sea to the southern and eastern coasts (Stone et al., 2020), otolith microchemical proxies of water constituents are dependent upon prolonged residence time within different water domains (Campana, 2005).

The highest migration rate calculated by BA3‐SNPs was from the Yellow Sea into the East Sea, even though such dispersal has to occur via the southern coast. This high connectivity may be associated with similar temperatures in the Yellow and East Seas. The second highest migration rate calculated by BA3‐SNPs was from the warmer southern coast to the cooler eastern coast. This was also the only eastward migration estimate with a 95% confidence limit including zero, likely due to the small sample size from the east coast population. Nevertheless, one of the two second‐generation east coast immigrants identified by BA3‐SNPs was an individual with south coast ancestry, suggesting the potential for some spread of thermally adaptive alleles into the East Sea.

Our data suggest that most dispersers are not successfully reproducing, as migration rate estimated from assignment tests (Tables S2–3) vastly exceeded that expected from levels of population differentiation and the number of second‐generation immigrants identified by BA3‐SNPs. We observed nine (BA3‐SNPs) to ten (GeneClass2) first‐generation dispersers, including eight mature adults (Figure 2), in 243 sampled fish. This corresponds to a migration rate of about 4%. In contrast, the identification of only two second‐generation immigrants (i.e., two reproductively successful immigrants) produces a migration rate of 0.8%. This estimate is almost identical to the long‐term estimate of *m* from *F_ST_*. Given an average between‐population *F_ST_* of 0.034 and an overall harmonic mean population size of *N_e_* = 909 individuals, we would expect a migration rate of 0.8% (*m* = 0.78%, $m\approx \frac{1}{4N_{e}}\left(\frac{1}{F_{\mathrm{ST}}}‐1\right)$). Although the assumptions of this approach are somewhat unrealistic (migration–drift equilibrium, infinite island model of migration, and equal *N_e_*/*N* ratios in migrant and resident populations; Whitlock & McCauley, 1999), estimates of *m* from *F_ST_* can be close to true values (Spies et al., 2018). Gene flow was therefore lower than expected from the frequency of dispersers, but short‐term demographic estimates from BA3‐SNPs correspond well to long‐term equilibrium estimates from *F_ST_*.

There are several potential scenarios which would explain the discrepancy between the number of dispersers and estimates of gene flow, with crucial implications for the potential for invasion of beneficial alleles from the southern, warm‐adapted population into northern populations stressed by increasing temperatures. First, countercurrent homing migration to spawn could maintain population structure despite larval and juvenile dispersal; the distance between the western and eastern Korean coasts is well within the seasonal migration distances of mature Pacific cod, which can reach over 1,000 km in the Bering Sea (Gustafson et al., 2000; Shimada & Kimura, 1994). Mature adult dispersers which did not return to source spawning locations may therefore be “skipped spawners” which do not reproduce in a given year (Rideout & Tomkiewicz, 2011) and so do not contribute to the local gene pool. Of the three mature migrants for which gonad weight was available, two were in the lower 50% of GSI spread at the site where they were sampled (Figure S4). Skipped spawning has been documented in other gadids, including Atlantic cod (Skjæraasen et al., 2012).

Second, local adaptation may limit the reproductive success of dispersers and thus reduce effective gene flow (Peterson et al., 2014). High correlation between temperature and allele frequencies suggested adaptive genetic divergence between peripheral populations around the Korean Peninsula, due at least in part to different thermal regimes during the spawning season. There is similarly strong evidence for temperature adaptation in Atlantic cod, where temperature has been linked to multiple anonymous SNP loci (Bradbury et al., 2010) and genetic variation at the *PanI* gene, which encodes for a membrane protein (Case et al., 2005). Climate change may weaken such barriers to gene flow around the Korean Peninsula caused by environmental, physical, and biological factors. The extent to which this occurs will partly depend on the degree of existing environmental heterogeneity (which is considerable; Rebstock & Kang, 2003) that persists under climate change, as the simultaneous spread of locally maladaptive alleles could outweigh the beneficial effects of temperature‐associated evolutionary rescue (Bourne et al., 2014).

Limits to gene flow from adaptive differentiation may also be aided by chromosomal inversions that facilitate local adaptation by reducing recombination rates (Wellenreuther & Bernatchez, 2018). In Atlantic cod, polymorphic chromosome inversions spanning over 10Mb have been found to facilitate and maintain genetic divergence between migratory and stationary ecotypes (Barth et al., 2017), inshore and offshore spawning populations (Barney et al., 2017), and fjord and oceanic populations that spawn sympatrically (Kirubakaran et al., 2016; Sodeland et al., 2016). Investigating patterns of linkage disequilibrium between peripheral populations of Pacific cod would reveal the extent to which similar mechanisms affect fine‐scale population differentiation in this species. Such barriers would likely persist even under a changing climate.

By showing the potential for the spread of adaptive alleles between peripheral populations around the Korean Peninsula, this research brings into question the potential for adaptive change in populations further north, at the core of the species’ range. From the eastern Korean coast, which received first‐ and second‐generation immigrants from the warmer, southern coast, the East Sea extends north to the Sea of Okhotsk via the Mamiya Strait/Strait of Tartary. Analysis of microsatellite and mitochondrial DNA has shown that Pacific cod in the East Sea are more highly connected to populations in these northern waters than to the Yellow Sea, the source of three first‐generation immigrants, and the highest contemporary immigration rate to the East Sea in our study. Microsatellite DNA showed samples from the eastern coast of the Korean Peninsula and the more northern Hokkaido, Japan, to be less differentiated than samples from the eastern and western coasts of the Korean Peninsula (*F_ST_* = 0.019 and 0.033, respectively; Kim et al., 2010). *F_ST_* calculated from mitochondrial DNA also showed greater differentiation between samples from the Korean Peninsula’s western and eastern coasts (*F_ST_* = 0.128) than between samples from the eastern Korean coast and sites in the north/northwestern Sea of Okhotsk (*F_ST_* = 0.029–0.056; Orlova et al., 2019). Further research may reveal whether existing gene flow is sufficient to export adaptive alleles from the Korean peripheral populations to more northern Pacific cod, or whether assisted gene flow (Aitken & Whitlock, 2013) should be attempted, keeping in mind potential risks associated with outbreeding depression and domestication selection in hatchery programs (Naish et al., 2008).

## 5 CONCLUSIONS

Notwithstanding specific mechanisms of dispersal and adaptation, our results strongly support the inherent conservation value of peripheral, isolated, and old populations at the southern edge of temperate marine species’ ranges (Hampe & Petit, 2005; Nadeau & Urban, 2019; Provan & Maggs, 2012). Distribution shifts may in many cases mask an irreversible loss of isolated populations, and with them a loss of genetic diversity that includes adaptive variation important for response to environmental change (Nadeau & Urban, 2019). Our results highlight the presence of adaptive alleles in small peripheral populations of Pacific cod and demonstrate contemporary migration from warm‐ to cold‐adapted populations, raising the question of whether gene flow of adaptive genetic variation from peripheral to core populations of Pacific cod may be possible. Peripheral populations should therefore be conserved and maintained as an important genetic resource, even if anthropogenic pressures require specific conservation measures. Further research into the ubiquity of these patterns in the marine realm, and the extent of gene flow from peripheral to core populations, is urgently needed.

## CONFLICT OF INTEREST

The authors declare no conflict of interest.

## AUTHOR CONTRIBUTIONS

**Mary C. Fisher:** Data curation (lead); formal analysis (equal); investigation (lead); methodology (equal); software (lead); validation (equal); visualization (lead); writing – original draft (lead); writing – review and editing (equal). **Thomas E. Helser:** Conceptualization (equal); funding acquisition (lead); project administration (equal); resources (equal); supervision (equal); writing – original draft (supporting); writing – review and editing (equal). **Sukyung Kang:** Conceptualization (equal); data curation (equal); funding acquisition (equal); resources (equal); writing – original draft (supporting); writing – review and editing (equal). **Woo Gwak:** Conceptualization (equal); data curation (equal); resources (equal); writing – original draft (supporting); writing – review and editing (equal). **Michael F. Canino:** Conceptualization (equal); writing – original draft (supporting); writing – review and editing (equal). **Lorenz Hauser:** Conceptualization (equal); formal analysis (equal); funding acquisition (lead); investigation (supporting); methodology (equal); project administration (equal); resources (equal); supervision (equal); validation (equal); writing – original draft (lead); writing – review and editing (equal).

## Supporting information

Supplementary Material

## Data Availability

Sequencing data have been deposited in the National Center for Biotechnology Information Sequence Read Archive (ncbi.nlm.nih.gov/sra; BioProject ID PRJNA786546). A file containing the final genotypes for all individuals is available from the Dryad Digital Repository (https://doi.org/10.5061/dryad.cjsxksn6r).
