# Supplementary material for: Genetic structure and dispersal in peripheral populations of a marine fish (Pacific cod, Gadus macrocephalus) and their importance for adaptation to climate change
Source: Ecol Evol. 2021 Dec 21;12(1):e8474. doi: 10.1002/ece3.8474 (PMC8794718; doi:10.1002/ece3.8474)
Supplement: Supplementary file 1 — Supplementary Material [file ECE3-12-e8474-s001.docx]

**Supplemental Information for**

**Genetic structure and dispersal in peripheral populations of a marine fish (Pacific cod, *Gadus macrocephalus*) and their importance for adaptation to climate change**

Mary C. Fisher*, Thomas E. Helser, Sukyung Kang, Wooseok Gwak, Michael F. Canino, Lorenz Hauser

* Corresponding Author | Email: [mfisher5@uw.edu](file:///C:\Users\mcf05\Downloads\mfisher5@uw.edu)

Table of Contents

**Figure S1.** Population assignment predictions for sampling sites, based on previous microsatellite DNA research**2**

**Figure S2.** Sea surface temperatures around the Korean Peninsula**3**

**Figure S3.** Pacific cod catch in South Korea coastal waters**4**

**Figure S4.** PCA and DAPC using only loci putatively under selection**5**

**Figure S5.** Boxplots of gonadosomatic index (GSI)**6**

**Figure S6.** Assignment success to population of origin**7**

**Figure S7.** Assignment success to collection of origin**8**

**Figure S8.** BA3-SNPs inferred posterior mean migration rates**9**

**Figure S9.** Counts of loci putatively under selection, by method**10**

**Table S1.** Pairwise *F_ST_* using only putatively neutral loci **11**

**Table S2.** Information on dispersers**12**

**Table S3.** Dispersers’ inferred proportional ancestry (Structure)**13**

**Table S4.** Dispersers’ assignment to population of origin (GeneClass)**14**

**Table S5.** BA3-SNPs inferred posterior mean migration rates**15**

**Table S6.** *N_e_* for each collection, with and without dispersers**16**

**Table S7.** Wilcoxon test on *F_is_* and *H_o_* between spawning seasons**17**

**Table S8.** Candidate outlier loci (Bayescan, OutFLANK)**18**

**Table S9.** Loci with allele frequencies correlated with temperature (Bayenv2)**19**

**Table S10.** Bayescan, OutFLANK loci aligned within annotated, protein-coding regions**20**

**Table S11.** Bayenv2 loci aligned within annotated, protein-coding regions**21-22**

**References23**


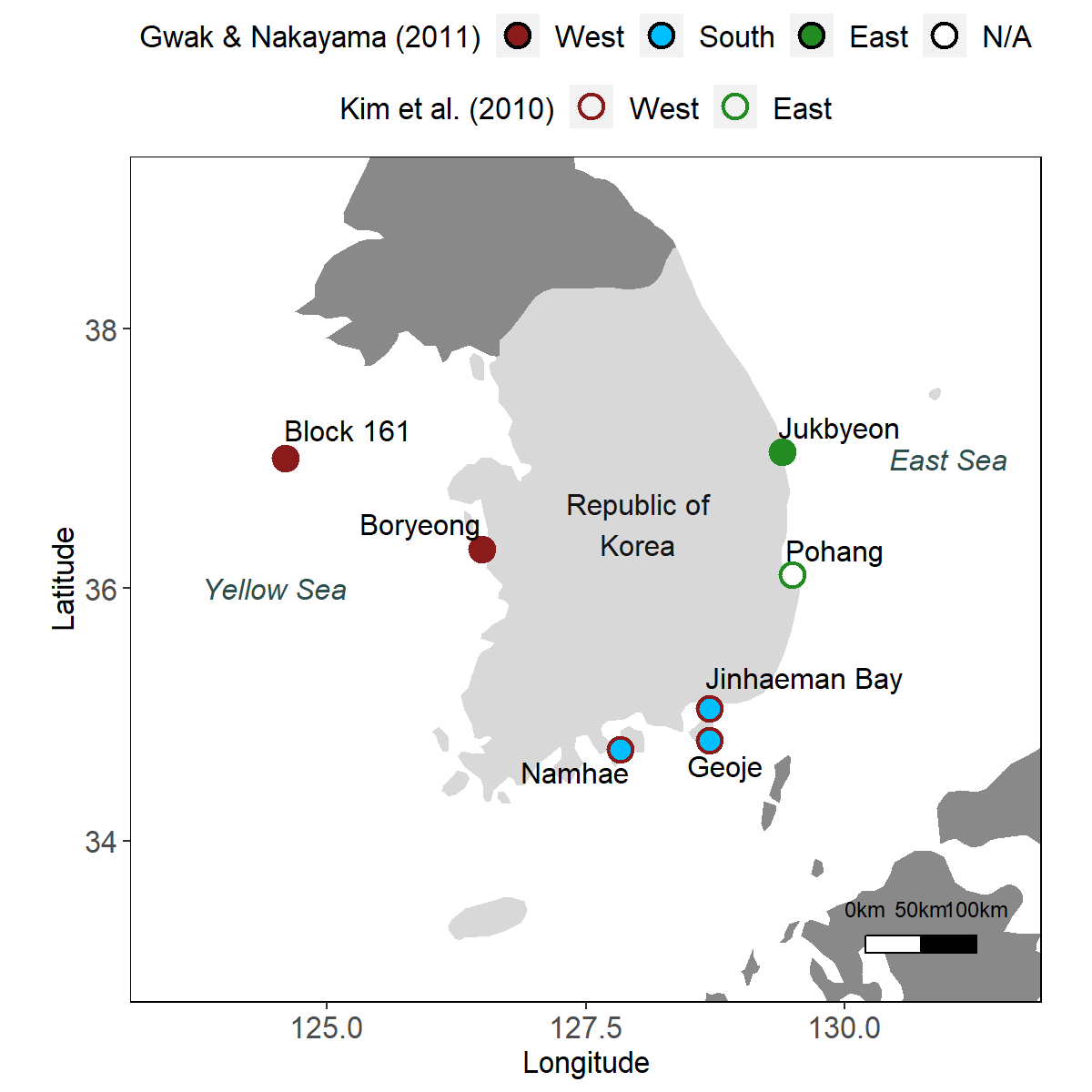


**Figure S1.**  Population assignment predictions for this study’s sampling sites, based on results from previous microsatellite DNA research focused on the Korean peninsula (Gwak & Nakayama, 2011; Kim, An, & Choi, 2010). Point fill represents the putative population according to the genetic structure described by Gwak & Nakayama (2011), and point outline according to Kim et al. (2010).

**
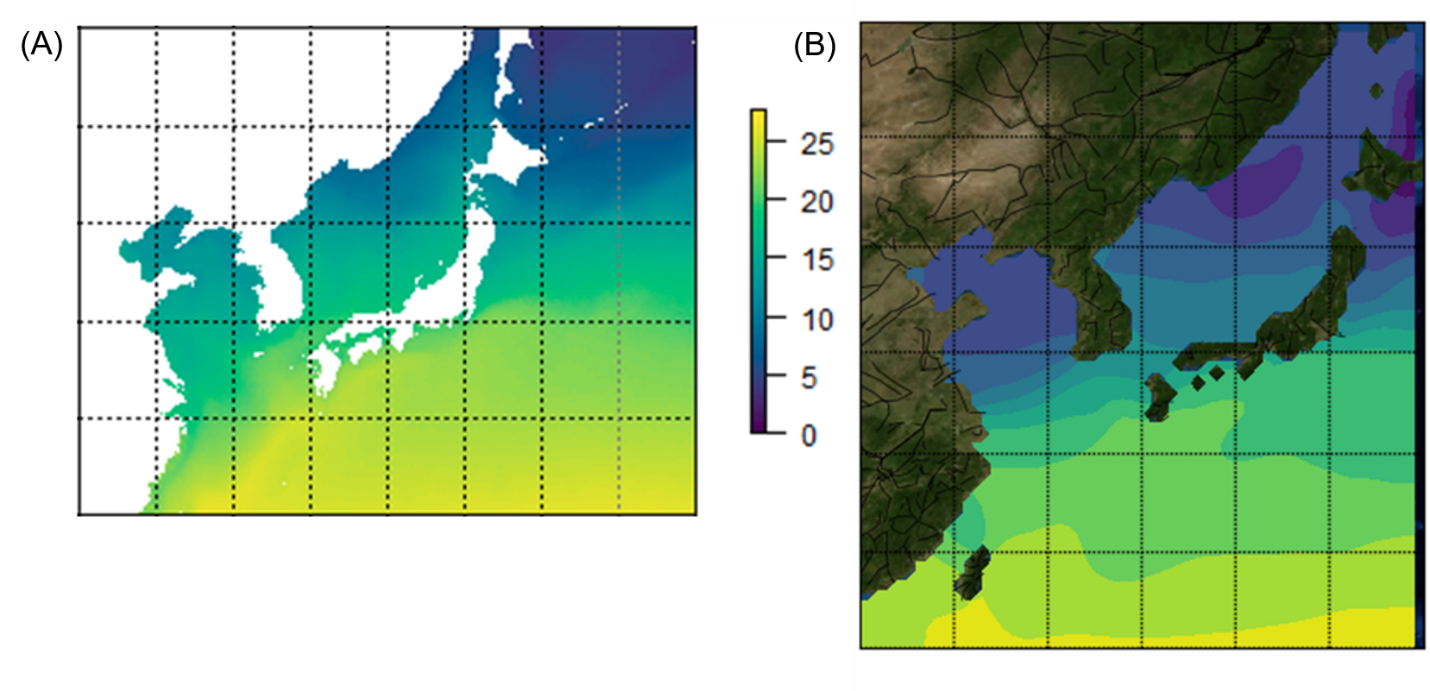
**

**Figure S2.** (A) Mean annual and (B) winter sea surface temperatures around the Korean Peninsula. Mean annual temperatures are drawn from the Bio-ORACLE data base (Tyberghein et al., 2012), v2.0 (Assis et al., 2018), at a 9.2km resolution (Assis et al., 2018; Tyberghein et al., 2012), and were one of the temperature datasets correlated with allele frequencies in Bayenv (Coop, Witonsky, Di Rienzo, & Pritchard, 2010). Winter (spawning period) temperatures are based on February temperature data, extracted from the Monthly Isopycnal / Mixed-layer Ocean Climatology (MIMOC) database, v2.2, at a 0.5° lateral resolution (Schmidtko, Johnson, & Lyman, 2013); the color scale indicates conservative temperature (TEOS-10) in degrees Celsius.


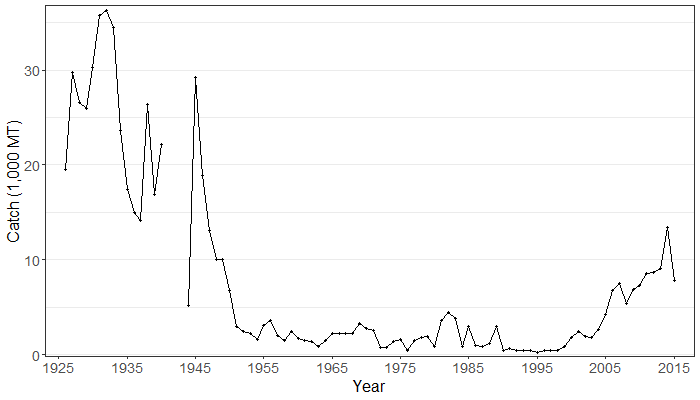


**Figure S3.** Pacific cod catch (in metric tons) in South Korea coastal waters, from 1926 through 2015. *Data provided by Dr. Sukyung Kang, National Institute of Fisheries Science.*

**
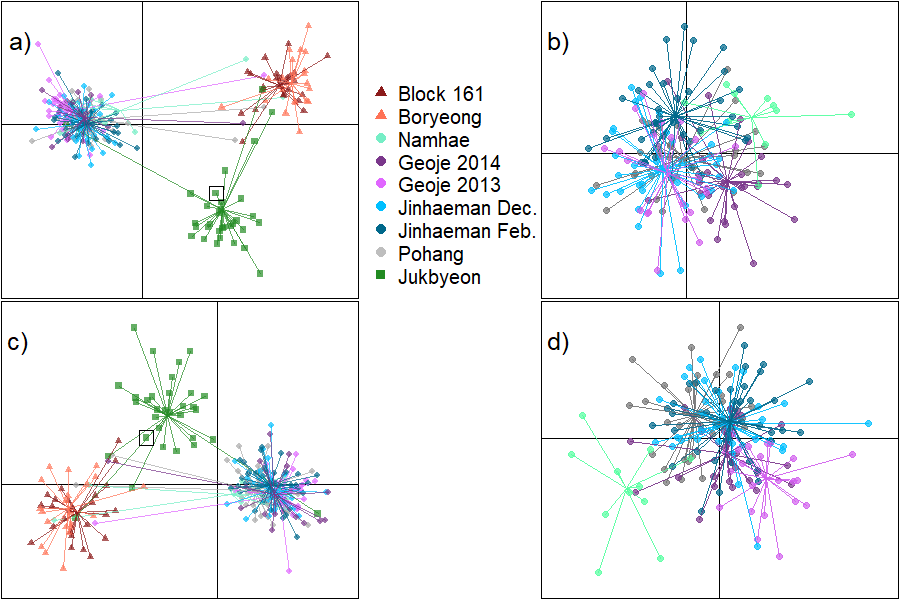
**

**Figure S4**. Principal component analysis of all collections (a,c) and discriminant analysis of principal components for just southern coast collections (b,d) using **(a,b)** loci identified as putatively under selection by one or more of OutFLANK, Bayescan (prior odds 10 – 10,000), and Bayenv (n=147); and **(c,d)** only loci identified by Bayenv as having allele frequencies correlated with temperature (n=87). The second-generation immigrant sampled at Jukbyeon that was only identified by BA3-SNPs is circled in black for (a,c).

**
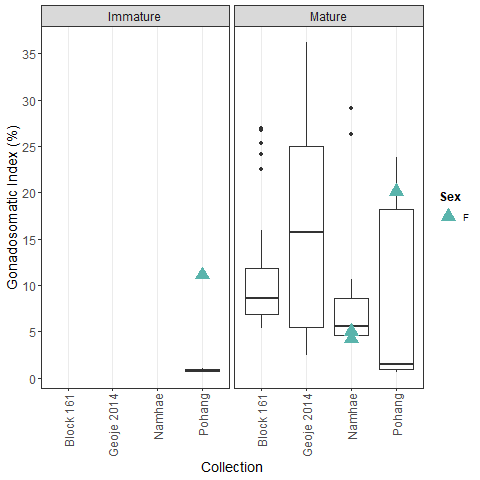
**

**Figure S5.** Boxplots of gonadosomatic index (GSI) for the four collections for which gonad weight was recorded for each individual. Maturity classification for each individual was determined according to sex-specific 50% length-at-maturity (see **Figure S1**). Overlaid triangles represent the gonadosomatic indices of dispersers for which gonad weight was available.

**
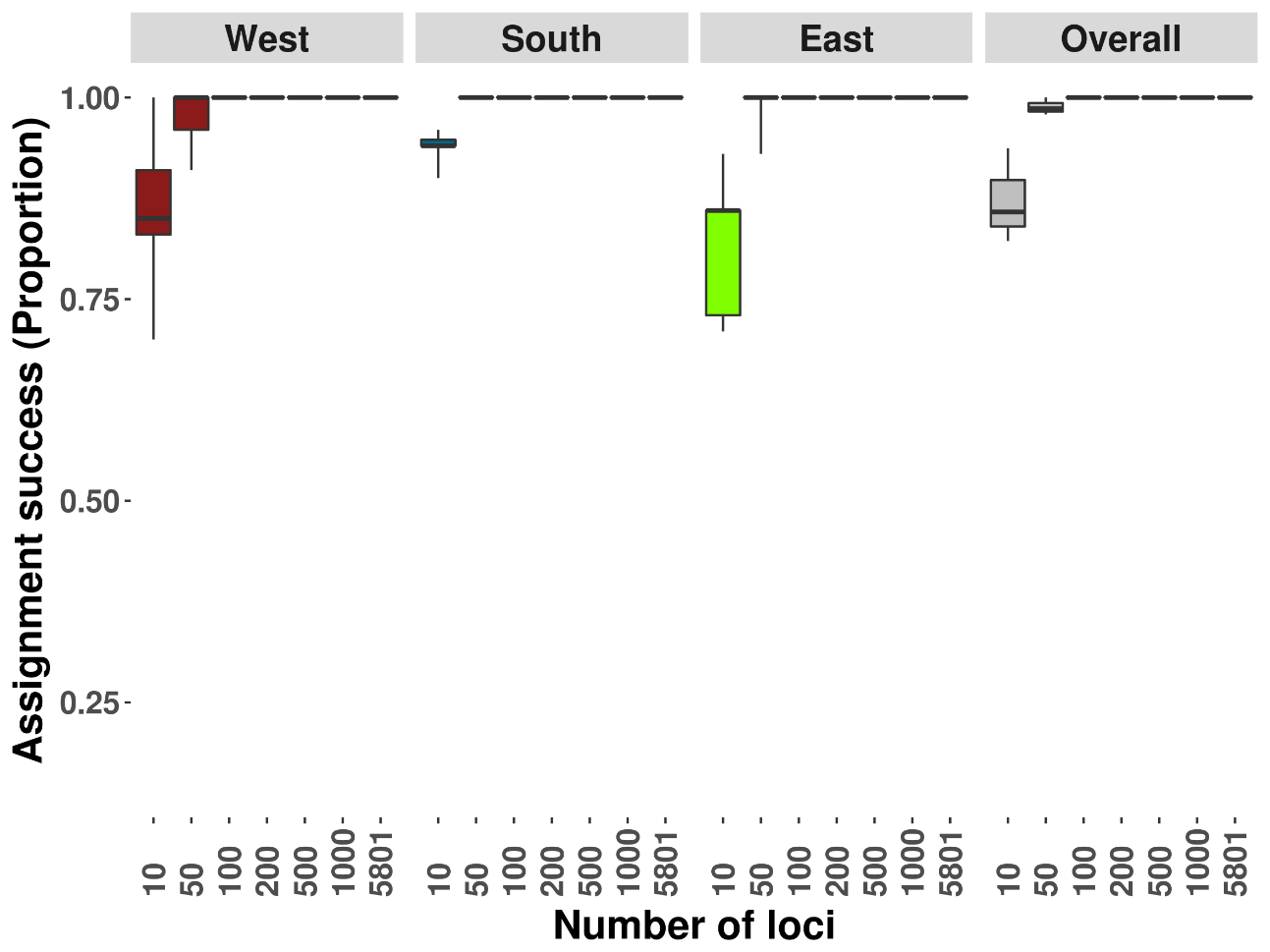
**

**Figure S6**. Assignment success (with standard error) when assigning individuals to population of origin. These results excluded dispersers. Assignment success is measured as the proportion of individuals sampled from each collection which were assigned back to that collection through genetic stock identification.


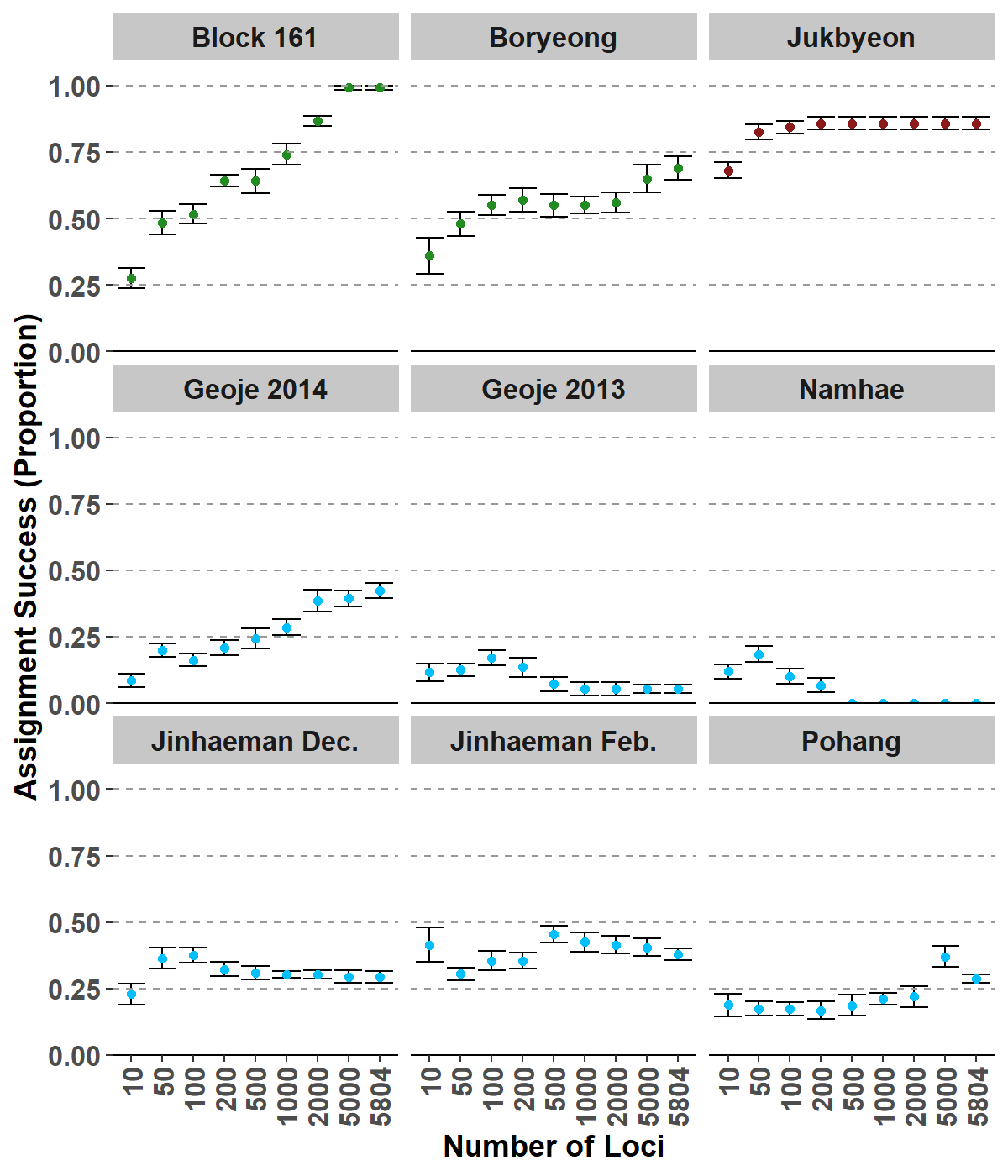


**Figure S7.** Assignment success (with standard error) when assigning individuals to collection of origin. These results excluded dispersers. Assignment success is measured as the proportion of individuals sampled from each collection which were assigned back to that collection through genetic stock identification.

**
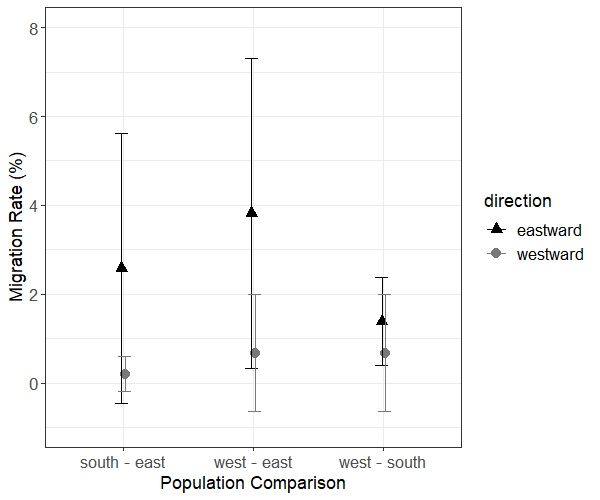
**

**Figure S8.** BA3-SNPs inferred posterior mean migration rates between coastal populations, with 95% confidence intervals. Confidence intervals were constructed from the standard deviation of the marginal posterior distribution per the *BayesAss Edition 3.0 User’s Manual* (Rannala, 2007).

**
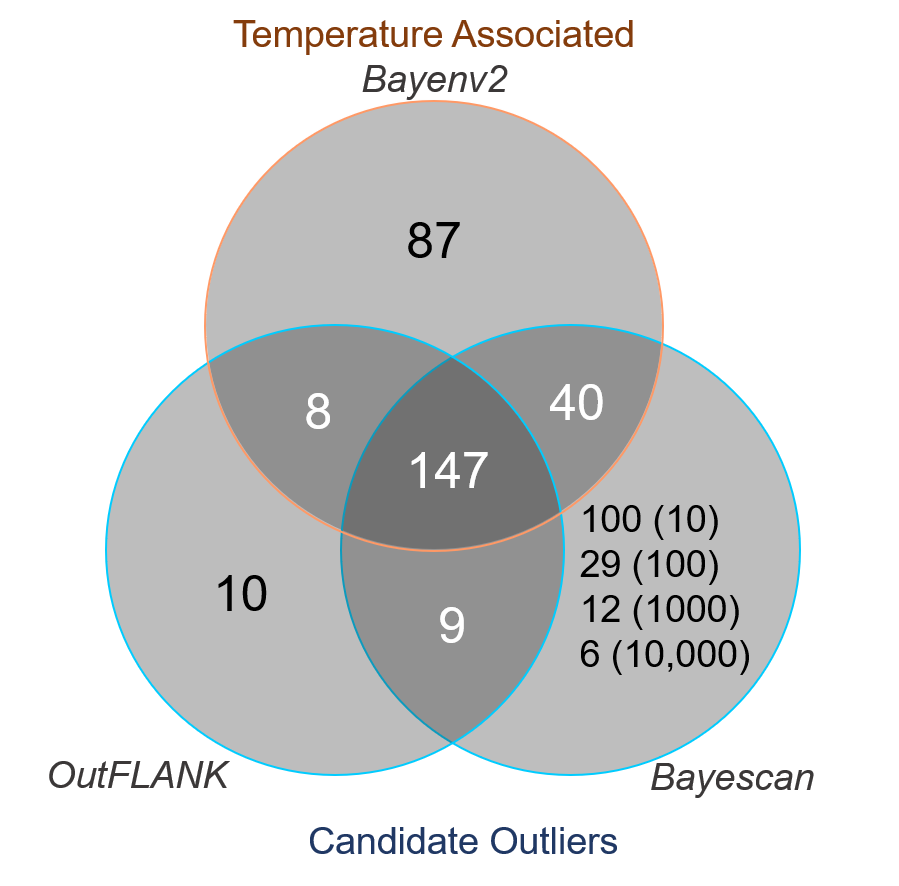
**

**Figure S9.** Diagram showing counts of loci putatively under selection, for each program used. For Bayescan, prior odds (1 in 10, 100, 1000, 10000) are specified in parentheses next to each locus count. Joint counts with Bayescan are for loci identified with prior odds of the neutral model set to 1 in 10.

**Table S1.** Pairwise *F_ST_* between collections, using only putatively neutral loci. We removed loci identified as under selection using OutFLANK or Bayescan (prior odds 10, 100, 1000, and 10000), or those identified in Bayenv2 as possessing allele frequencies decisively associated with water temperature. Statistically significant differentiation (p < 0.05) is indicated with (*) for Fisher’s exact test. For pairwise *F_ST_* calculated using all loci, see Table 1.

|  | **Block 161** | **Boryeong** | **Namhae** | **Geoje 2014** | **Geoje 2013** | **Jinhaeman Dec.** | **Jinhaeman Feb.** | **Pohang** |
| --- | --- | --- | --- | --- | --- | --- | --- | --- |
| **Boryeong** | 0.0028 |  |  |  |  |  |  |  |
| **Namhae** | 0.0255* | 0.0257* |  |  |  |  |  |  |
| **Geoje 2014** | 0.0387* | 0.0407* | 0.0030 |  |  |  |  |  |
| **Geoje 2013** | 0.0366* | 0.0382* | 0.0047 | 0.0031 |  |  |  |  |
| **Jinhaeman Dec.** | 0.0405* | 0.0422* | 0.0037 | 0.0031 | 0.0010 |  |  |  |
| **Jinhaeman Feb.** | 0.0372* | 0.0389* | 0.0018 | 0.0025 | 0.0007 | 0.0002 |  |  |
| **Pohang** | 0.0349* | 0.0364* | 0.0022 | 0.0022 | 0.0013 | 0.0016 | 0.0008 |  |
| **Jukbyeon** | 0.0275* | 0.0263* | 0.0305* | 0.0403* | 0.0393* | 0.0418* | 0.0389* | 0.0375* |

**Table S2.** Information for individuals which were sampled in one region and genetically assigned to a different region (“dispersers”). Percent missing data was calculated as proportion of loci not genotyped in the individual. DNA and RAD plate proximity describe whether individuals from the population to which the sample was assigned was close to (i.e., located within one well next to) the sample during DNA extraction or RAD library preparation.

|  | **Sampling Site** | **Collection Date** | **Sample ID** | ***H_o_*** | **% Missing Data** | **Related Samples (ML relate)** | **DNA Plate Proximity** | **RAD Plate Proximity** | **DNA Quality** |
| --- | --- | --- | --- | --- | --- | --- | --- | --- | --- |
| 1 | Namhae | 2/10/2015 | 22 | 0.177 | 0.8 | None | None | None | Good |
| 2 | Namhae | 2/10/2015 | 30 | 0.185 | 0.3 | None | None | Yes | Good |
| 3 | Geoje | 1/23/2015 | 06 | 0.215 | 1.2 | None | None | None | Good |
| 4 | Geoje | 2/4/2014 | 06 | 0.150 | 6.7 | None | None | None | Good |
| 5 | Pohang | 1/7/2015 | 11 | 0.176 | 1.0 | None | None | None | Good |
| 6 | Pohang | 2/5/2015 | 06 | 0.210 | 0.9 | None | None | None | Good |
| 7 | Jukbyeon | 12/10/2007 | 01 | 0.153 | 3.7 | None | Yes | None | Good |
| 8 | Jukbyeon | 12/10/2007 | 13 | 0.179 | 3.8 | None | None | None | Good |
| 9 | Jukbyeon | 12/10/2007 | 31 | 0.181 | 0.8 | Half-sib with Jinhaeman Bay sample | None | Yes (not related sample) | Slightly Degraded |
| 10 | Jukbyeon | 12/10/2007 | 33 | 0.179 | 0.9 | None | None | Yes | Good |

**Table S3.** Inferred proportional ancestry for dispersers identified in the PCA (Figure 3). Cluster 1 consisted entirely of western coast samples, Cluster 2 of southern/southeastern coast samples, and Cluster 3 of eastern coast samples. Proportions are averages of three replicate runs in STRUCTURE. A (*) indicates the cluster with the highest proportional ancestry for the disperser, and a (^+^) the cluster to which the other individuals from the same sampling site were assigned.

|  |  |  |  | Inferred Ancestry | | |
| --- | --- | --- | --- | --- | --- | --- |
|  | **Sampling Site** | **Collection Date** | **Sample ID** | **Cluster 1** | **Cluster 2** | **Cluster 3** |
| 1 | Namhae | 2/10/2015 | 22 | 0.950* | 0.043^+^ | 0.009 |
| 2 | Namhae | 2/10/2015 | 30 | 0.971* | 0.026^+^ | 0.002 |
| 3 | Geoje | 1/23/2015 | 06 | 0.924* | 0.072^+^ | 0.004 |
| 4 | Geoje | 2/4/2014 | 06 | 0.991* | 0.006^+^ | 0.004 |
| 5 | Pohang | 1/7/2015 | 11 | 0.937* | 0.032^+^ | 0.031 |
| 6 | Pohang | 2/5/2015 | 06 | 0.833* | 0.142^+^ | 0.025 |
| 7 | Jukbyeon | 12/10/2007 | 01 | 0.907* | 0.018 | 0.075^+^ |
| 8 | Jukbyeon | 12/10/2007 | 13 | 0.980* | 0.006 | 0.015^+^ |
| 9 | Jukbyeon | 12/10/2007 | 31 | 0.001 | 0.994* | 0.004^+^ |
| 10 | Jukbyeon | 12/10/2007 | 33 | 0.983* | 0.004 | 0.013^+^ |

**Table S4.** Assignment probability to population of origin (GeneClass) and generation (BA3-SNPs; all posterior probabilities 1.0) for dispersers.

|  | Sampling Site | Collection Date | Sample ID | Population of Origin | Probability (GeneClass) | Generation (BA3-SNPs) |
| --- | --- | --- | --- | --- | --- | --- |
| 1 | Namhae | 2/10/2015 | 22 | West | 1.0 | F1 |
| 2 | Namhae | 2/10/2015 | 30 | West | 1.0 | F1 |
| 3 | Geoje | 1/23/2015 | 06 | West | 1.0 | F2 |
| 4 | Geoje | 2/4/2014 | 06 | West | 1.0 | F1 |
| 5 | Pohang | 1/7/2015 | 11 | West | 1.0 | F1 |
| 6 | Pohang | 2/5/2015 | 06 | West | 1.0 | F1 |
| 7 | Jukbyeon | 12/10/2007 | 01 | West | 1.0 | F1 |
| 8 | Jukbyeon | 12/10/2007 | 13 | West | 1.0 | F1 |
| 9 | Jukbyeon | 12/10/2007 | 31 | South | 1.0 | F1 |
| 10 | Jukbyeon | 12/10/2007 | 33 | West | 1.0 | F1 |

**Table S5.** Inferred posterior mean migration rates with 95% confidence intervals from BA3-SNPs. Confidence intervals were constructed from the standard deviation of the marginal posterior distribution per the *BayesAss Edition 3.0 User’s Manual* (Rannala, 2007). The number of observed dispersers (from the PCA and Structure analyses) is reported for each pair of populations.

| Direction | Source Population | Receiving Population | Mean (95% Confidence Interval) | Observed Dispersers |
| --- | --- | --- | --- | --- |
| Eastward | West | South | 0.0139 (0.0239, 0. 0039) | 6 |
|  | West | East | 0.0382 (0.0731, 0. 0033) | 3 |
|  | South | East | 0.0258 (0.0562, -0. 0046) | 1 |
| Westward | East | South | 0.0020 (0.0059, -0.0019) | 0 |
|  | East | West | 0.0068 (0.0199, -0.0063) | 0 |
|  | South | West | 0.0068 (0.0199, -0.0063) | 0 |

**Table S6.** Effective population sizes *not corrected for linkage* for each collection, before and after detected dispersers (N_m_) were removed from the data. *N_e_* is reported for each of the lowest minor allele frequencies used (0.01, 0.02, 0.05), with 95% confidence intervals given in parentheses. There were too few samples from Namhae to provide an accurate estimate of *N_e_* (n=12).

|  |  | Dispersers Included | | |  | Dispersers Removed | | |
| --- | --- | --- | --- | --- | --- | --- | --- | --- |
| Sampling Site | Spawning Season | N_e_ (0.01) | N_e_ (0.02) | N_e_ (0.05) | N_m_ | Recalc.  N_e_ (0.01) | Recalc.  N_e_ (0.02) | Recalc.  N_e_ (0.05) |
| Block 161 | 2015 | 943  (838-1078) | 943  (838- 1078) | 541  (492-602) | 0 |  |  |  |
| Boryeong | 2007 | 2051  (1564-2987) | 2051  (1564-2987) | 1394  (1077-1976) | 0 |  |  |  |
| Namhae | 2014 | - | - | 490  (388-664) | 2 | - | - | - |
| Geoje | 2014 | 436  (415-459) | 911  (814-1033) | 914  (805-1058) | 1 | 1083  (959-1243) | 942  (834-1081) | 819  (725-940) |
|  | 2013 | 447  (415-484) | 447  (415-484) | 427  (388-474) | 1 | 938  (802-1130) | 938  (802-1130) | 495  (442-561) |
| Jinhaeman Bay | 2007, Dec. | 236  (231-241) | 186  (183-190) | 148  (145-151) | 0 |  |  |  |
|  | 2007, Feb. | 654  (623-689) | 685  (646-729) | 605  (567-648) | 0 |  |  |  |
| Pohang | 2014 | 410  (395-427) | 934  (849-1038) | 1108  (965-1300) | 2 | 2307  (1837-3099) | 1640  (1357-2071) | 1553  (1269-2000) |
| Jukbyeon | 2007 | 653  (615-696) | 789  (727-861) | 1007  (887-1164) | 4 | 1074  (958-1223) | 1078  (947-1249) | 1306  (1088-1632) |

**Table S7.** Results from the Wilcoxon rank sum test for statistically significant differences in *F_is_* and observed heterozygosity (*H_o_*) between **(a)** the 2007 and 2015 spawning seasons in the western population, and **(b)** the 2007 and 2013 spawning seasons in the southern population, and **(c)** the 2013 and 2014 spawning seasons in the southern population.

|  | **Population** | **Season 1** | **Season 2** | **Variable** | **Test statistic (W)** | **p-value** |
| --- | --- | --- | --- | --- | --- | --- |
| **(a)** | West | 2007 | 2015 | *F_is_* | 10682000 | 1.24 x 10^-6^ |
|  |  |  |  | *H_o_* | 11733000 | 0.003 |
| **(b)** | South | 2007 | 2013 | *F_is_* | 9735100 | 5.57 x 10^-12^ |
|  |  |  |  | *H_o_* | 10183000 | 6.91 x 10^-4^ |
| **(c)** | South | 2013 | 2014 | *F_is_* | 11950000 | < 2.20 x 10^-16^ |
|  |  |  |  | *H_o_* | 9687500 | 1.12 x 10^-11^ |

**Table S8.** Outlier loci identified in both Bayescan (given priors ‘p.’) and OutFLANK, with p-values (corrected for false discovery rate). Loci listed above the line were detected in full dataset; the locus listed below the line was detected as a candidate outlier locus only between sampling sites within the southern coast population.

|  |  | *q value* | | | | |
| --- | --- | --- | --- | --- | --- | --- |
| Data set | **Locus ID** | **OutFLANK** | **Bayescan p. 10** | **Bayescan p. 100** | **Bayescan p. 1K** | **Bayescan p. 10K** |
| All Data | 10203 | 0.034 | 0 | 0 | 1.5 x 10^-5^ | 0.0070 |
|  | 14546 | 0.0057 | 0 | 0 | 5 x 10^-5^ | 0.0008 |
|  | 19221 | 0.0008 | 0 | 0 | 0 | 0 |
|  | 2694 | 0.0123 | 0 | 0 | 7.5 x 10^-5^ | 0.0039 |
|  | 1904 | 0.0319 | 0 | 0.0002 | 0.0055 |  |
|  | 18723 | 0.0123 | 0.0004 | 0.0064 |  |  |
|  | 3699 | 0.0183 | 0.0001 | 0.0013 |  |  |
|  | 2606 | 0.0340 | 0.0029 |  |  |  |
|  | 3405 | 0.0144 | 0.0041 |  |  |  |
| Southern Population | 24927 | 0.466526 | 5 x 10^-5^ | 0.0005 | 0.0214 |  |

**Table S9.** Total number of loci with allele frequencies definitively correlated with each of the given temperature variables, and the number of those loci which had the highest or lowest allele frequency in the southern population. For loci counts across all variables, only the first occurrence of each locus is counted.

| Variable | Total Loci | Highest South | Lowest South |
| --- | --- | --- | --- |
| Mean Sea Surface Temperature (SST) | 64 | 30 | 30 |
| Maximum Sea Surface Temperature (SST) | 3 | 1 | 0 |
| Mean Temperature at Maximum Depth | 9 | 3 | 3 |
| Minimum Temperature at Maximum Depth | 48 | 21 | 0 |
| *Across All Variables (Unique Loci)* | *87* | *38* | *36* |

**Table S10.** Biological functions of candidate outlier loci (identified by Bayescan, OutFLANK) that aligned to annotated, protein-coding regions in the Atlantic cod genome. The last column provides the number of candidate outlier loci on each linkage group, and total number and percent of aligned loci associated with each general function; the sum of percentages is above 100% because one locus aligned to two protein-coding gene regions associated with different general functions (CTTNBP2 and CFTR). Gene functions drawn from UniProtKB (The UniProt Consortium, 2018).

| **General Function** | **Specific Functions** | **Genes** | **Linkage Group** | **No. (%) Aligned Loci** |
| --- | --- | --- | --- | --- |
| Sensory | Pheromone response | Vmn2r26 |  | 1 (4%) |
|  |  |  | 16 | 1 |
| Reproduction & Early Development | Spinogenesis; sperm motility and sperm flagellar assembly; cell division, growth, and differentiation; oocyte growth and survival; embryogenesis | Kmtb2a/b, DHX38, PLEKHA7, CTTNBP2, Dnah2, BPC3 |  | 7 (29%) |
|  |  |  | 9 | 1 |
|  |  |  | 10 | 1 |
|  |  |  | 11 | 1 |
|  |  |  | 14 | 2 |
|  |  |  | 16 | 1 |
|  |  |  | 17 | 1 |
| Immune | Innate immune system signal transduction; exocytosis in lymphosites and granule maturation, docking at immunologic synapse | FRMPD3; Unc13d |  | 2 (8%) |
|  |  |  | 10 | 1 |
|  |  |  | 18 | 1 |
| Other Cellular Functions | fluid homeostasis, ion and water transport; cellular response to hypoxia; metal ion binding; DNA repair; protein synthesis, sorting, and modification; plasmalemma repair and regeneration of skeletal muscle | Col26a1, ADD2, CFTR, TMEM245, DDB1, Myof, Rbsn, MRPS25, slu7, GOLIM4, Fndc1 |  | 10 (42%) |
|  |  |  | 1 | 2 |
|  |  |  | 6 | 2 |
|  |  |  | 9 | 1 |
|  |  |  | 10 | 1 |
|  |  |  | 11 | 1 |
|  |  |  | 14 | 1 |
|  |  |  | 16 | 1 |
|  |  |  | 17 | 1 |
|  |  |  | 21 | 1 |
| Unknown | NA | NA | 9, 11, 17 | 5 (21%) |

**Table S11.** Biological functions of loci with decisive associations with the given temperature variables, and the Atlantic cod linkage groups to which they aligned. The last column provides the number of loci on each linkage group, and total number and percent of aligned loci associated with each general function; the sum of percentages is above 100% because one gene was associated with two general functions (gdf6b; sensory, reproduction & early development). Gene functions drawn from UniProtKB (The UniProt Consortium, 2018).

| **General Function** | **Environmental Variable** | **Specific Functions** | **Genes** | **Linkage Group** | **No. Aligned Loci** |
| --- | --- | --- | --- | --- | --- |
| Sensory | Mean SST | Generating circadian rhythm; retinal development | ROCK2, gdf6b |  | 3 (7%) |
|  |  |  |  | 21 | 2 |
|  |  |  |  | 22 | 1 |
| Reproduction & Early Development | Mean & Max. SST, Mean & Min. Temperature at Depth | Biogenesis and maintenance of zonula adherens; Notch signaling pathway; cytokinesis, cell growth, and differentiation; regulation of spermatogenesis; cell-cell communication during embryonic development; extraembryonic development; cell division and polarization; Synapse and central nervous system development | PLEKHA7, PARD6B, kdelc1, RACGAP1, LRP4, DHX38, UBR5, Kmt2a/b, Sema4d, Cit, plekhh1, Ptprq, gdf6b |  | 14 (31%) |
|  |  |  |  | 1 | 1 |
|  |  |  |  | 5 | 1 |
|  |  |  |  | 9 | 1 |
|  |  |  |  | 11 | 3 |
|  |  |  |  | 13 | 1 |
|  |  |  |  | 14 | 3 |
|  |  |  |  | 16 | 1 |
|  |  |  |  | 19 | 1 |
|  |  |  |  | 20 | 1 |
|  |  |  |  | 22 | 1 |
| Immune | Mean SST,  Mean & Min. Temperature at Depth | T-cell development; induction of B-cells; DNA virus-triggered and CGAS-mediated innate immune response | clptm1, Sema4d, TMPO, Zdhhc1 |  | 4 (9%) |
|  |  |  |  | 9 | 1 |
|  |  |  |  | 14 | 1 |
|  |  |  |  | 16 | 1 |
|  |  |  |  | 19 | 1 |
| Other Cellular Functions | Mean SST,  Mean & Min. Temperature at Depth | Protein assembly; protein modification & metabolism; chromatin organization; cell attachment and migration; gene expression and cellular signaling; organelle transport; DNA repair | API5, Agl3 TMEM255B, TMPO, Antxr1, ADCY2, CSMD3, DCN, Trim3, ADD2, Senp6 |  | 11 (24%) |
|  |  |  |  | 6 | 2 |
|  |  |  |  | 7 | 1 |
|  |  |  |  | 8 | 1 |
|  |  |  |  | 9 | 2 |
|  |  |  |  | 11 | 1 |
|  |  |  |  | 14 | 1 |
|  |  |  |  | 16 | 1 |
|  |  |  |  | 19 | 1 |
|  |  |  |  | 21 | 1 |
| Unknown | Mean SST,  Min. Temperature at Depth | NA | NA | 3,4,8,9,10,  13,16,17,19  21,22 | 14 (31%) |

**References**

Gwak, W., & Nakayama, K. (2011). Genetic variation and population structure of the Pacific cod *Gadus macrocephalus* in Korean waters revealed by mtDNA and msDNA markers. *Fisheries Science, 77*(6), 945-952.

Kim, M.J., An, H.S., & Choi, K.H. (2010). Genetic characteristics of Pacific cod populations in Korea based on microsatellite markers. *Fisheries Science, 76*(4), 595-603.

Tyberghein, L., Verbruggen, H., Pauly, K., Troupin, C., Mineur, F., & De Clerck, O. (2012). Bio-ORACLE: a global environmental dataset for marine species distribution modelling. *Global Ecology and Biogeography, 21*(2), 272-281.

Assis, J., Tyberghein, L., Bosch, S., Verbruggen, H., Serrão, E. A., & De Clerck, O. (2018). Bio- ORACLE v2.0: Extending marine data layers for bioclimatic modelling. *Global Ecology and Biogeography, 27*(3), 277-284.

Coop, G., Witonsky, D., Di Rienzo, A., & Pritchard, J. K. (2010). Using environmental correlations to identify loci underlying local adaptation. *Genetics, 185*(4), 1411.

Schmidtko, S., Johnson, G. C., & Lyman, J. M. (2013). MIMOC: A global monthly isopycnal upper‐ocean climatology with mixed layers. *Journal of Geophysical Research: Oceans, 118*(4), 1658-1672.

Rannala, B. (2007). *BayesAss Edition 3.0 User’s Manual [Last Updated 28 Sept 2015].* Davis, CA: University of California, Davis.

The UniProt Consortium (2018). UniProt: the universal protein knowledgebase. *Nuclear Acids Resources, 46*.
